# Mitogenic Signals Stimulate the CREB Coactivator CRTC3 through PP2A Recruitment

**DOI:** 10.1016/j.isci.2018.12.012

**Published:** 2018-12-19

**Authors:** Tim Sonntag, Jelena Ostojić, Joan M. Vaughan, James J. Moresco, Young-Sil Yoon, John R. Yates, Marc Montminy

**Affiliations:** 1 Clayton Foundation Laboratories for Peptide Biology, The Salk Institute for Biological Studies, La Jolla, CA 92037, USA; 2 Department of Molecular Medicine, The Scripps Research Institute, La Jolla, CA 92037, USA

**Keywords:** Biological Sciences, Biochemistry, Molecular Biology, Cell Biology

## Abstract

The second messenger 3′,5′-cyclic adenosine monophosphate (cAMP) stimulates gene expression via the cAMP-regulated transcriptional coactivator (CRTC) family of cAMP response element-binding protein coactivators. In the basal state, CRTCs are phosphorylated by salt-inducible kinases (SIKs) and sequestered in the cytoplasm by 14-3-3 proteins. cAMP signaling inhibits the SIKs, leading to CRTC dephosphorylation and nuclear translocation. Here we show that although all CRTCs are regulated by SIKs, their interactions with Ser/Thr-specific protein phosphatases are distinct. CRTC1 and CRTC2 associate selectively with the calcium-dependent phosphatase calcineurin, whereas CRTC3 interacts with B55 PP2A holoenzymes via a conserved PP2A-binding region (amino acids 380–401). CRTC3-PP2A complex formation was induced by phosphorylation of CRTC3 at S391, facilitating the subsequent activation of CRTC3 by dephosphorylation at 14-3-3 binding sites. As stimulation of mitogenic pathways promoted S391 phosphorylation via the activation of ERKs and CDKs, our results demonstrate how a ubiquitous phosphatase enables cross talk between growth factor and cAMP signaling pathways at the level of a transcriptional coactivator.

## Highlights

• The mammalian CRTC family is distinctly regulated by protein phosphatases
• CRTC1 and CRTC2 bind to calcineurin, and PP2A is recruited to CRTC3
• CRTC3-PP2A complex formation is mediated by phosphorylation at S391 of CRTC3
• Mitogenic signals induce S391 phosphorylation via MAPKs and CDKs

## Graphical Abstract

**Figure:**
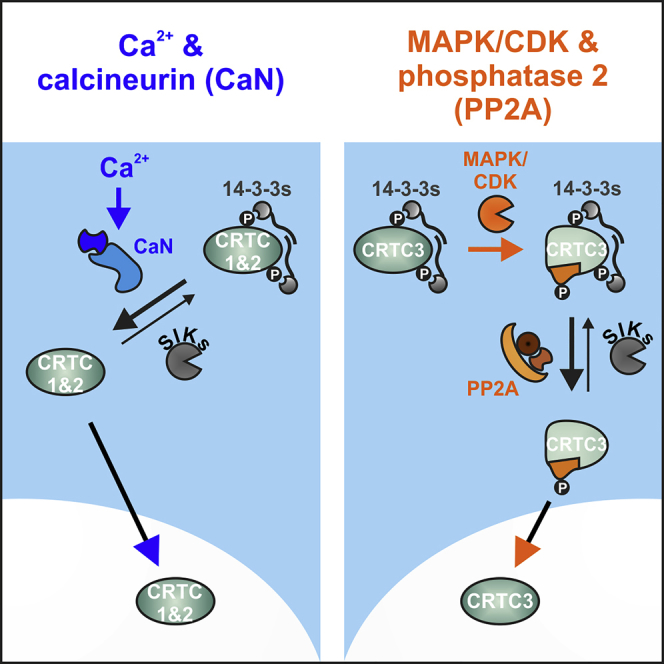

## Introduction

The second messenger 3′,5′-cyclic adenosine monophosphate (cAMP) is a potent driver of cellular responses to hormonal and environmental cues (Montminy, 1997). cAMP mediates the activation of the protein kinase A (PKA) holoenzyme (Taylor et al., 2012), which subsequently phosphorylates cellular substrates (Shabb, 2001). The transcriptional response to cAMP proceeds via the PKA-mediated phosphorylation of cAMP response element (CRE)-binding protein (CREB) family members (CREB1, ATF1, and CREM) (Mayr and Montminy, 2001). Phosphorylation of CREB at S133 induces a conformational change that enables the association of CREB with the histone acetyltransferases CREB-binding protein (CBP) and p300 (Parker et al., 1996). In parallel, cAMP also stimulates the association of CREB with the family of cAMP-regulated transcriptional coactivators (CRTCs, CRTC1–3) over relevant genomic CREB-binding sites (CRE sites) (Altarejos and Montminy, 2011).

Under basal conditions 5′ AMP-activated protein kinase family members, most notably the salt-inducible kinases (SIKs, SIK1–3), sequester the three CRTCs in the cytoplasm by phosphorylation at conserved sites, which promotes phosphorylation-dependent 14-3-3 protein binding (Sonntag et al., 2017, Wein et al., 2018). cAMP stimulation triggers the PKA-mediated phosphorylation and inhibition of SIK1–3 (Sonntag et al., 2018), leading to the dephosphorylation of CRTCs, which shuttle to the nucleus and drive cAMP/CREB target gene expression (Wein et al., 2018).

In addition to their regulation by SIKs, CRTCs are also controlled by calcium signaling via the Ca^2+^/calmodulin-dependent protein phosphatase calcineurin (CaN) (Perrino et al., 1995), which binds to and dephosphorylates the CRTCs at 14-3-3 binding sites (Altarejos and Montminy, 2011). The most abundant cellular phosphatases protein phosphatase 1 (PP1) and PP2A also appear to stimulate the dephosphorylation of CRTC2 (Shi, 2009, Uebi et al., 2010), although the context in which these ubiquitous enzymes regulate CRTC activities is unclear.

Multiple CRTC family members are co-expressed in mammalian tissues: CRTC1 and CRTC2 are produced in the brain (Altarejos et al., 2008, Jeanneteau et al., 2012) and pancreatic islets (Eberhard et al., 2013, Malm et al., 2016), for example, whereas CRTC2 and CRTC3 are found in fat (Henriksson et al., 2015, Song et al., 2010), liver (Koo et al., 2005, Patel et al., 2014), and bone marrow-derived immune cells (Kim et al., 2017, MacKenzie et al., 2013). Nevertheless, individual CRTCs appear to execute dominant roles in certain tissues, with CRTC1 controlling memory (Nonaka et al., 2014) and behavior (Breuillaud et al., 2012), entraining the circadian clock (Jagannath et al., 2013), and promoting energy expenditure (Altarejos et al., 2008). By contrast, CRTC2 regulates liver gluconeogenesis (Koo et al., 2005) and beta-cell viability (Blanchet et al., 2015, Eberhard et al., 2013), whereas CRTC3 promotes adipose tissue (Song et al., 2010) and anti-inflammatory macrophage function (MacKenzie et al., 2013). However, it is unknown whether these differences reflect the relative abundance or distinct activation properties of CRTC family members in relevant tissues.

Based on the importance of CRTC dephosphorylation for transcriptional activation, we systematically examined the relative association of individual CRTC family members across the mammalian complement of Ser/Thr-specific protein phosphatases by mass spectrometry. We identified selective interactions of CRTC1/2 with CaN and uncovered an unexpected role for PP2A in binding to and activating CRTC3 in response to extracellular signals. These results point to a novel role for PP2A in mediating effects of growth factor cues on the selective activation of a distinct CRTC family member.

## Results

### Selective Binding of Ser/Thr-Specific Protein Phosphatases to CRTC Family Members

**Figure 1:**
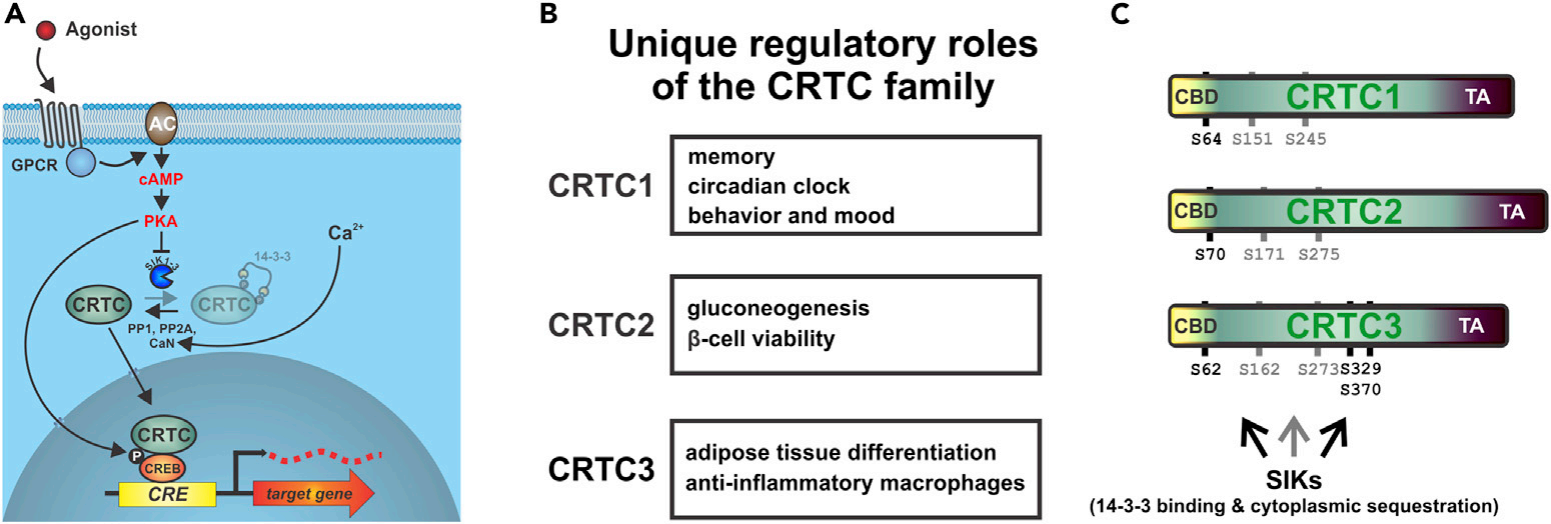
Common Regulation and Unique Functions of the CRTC Family (A) Upon activation of G protein-coupled receptors (GPCRs), cells generate the second messenger cAMP via adenylyl cyclases (ACs). cAMP induces PKA activity, which upon phosphorylation activates CREB and inactivates the SIKs. Correspondingly, CRTCs are dephosphorylated by the phosphatases PP1 and PP2A and the calcium-responsive CaN, leading to the loss of 14-3-3 protein binding and nuclear translocation. Inside the nucleus, CRTCs bind to CREB over relevant genomic cAMP response element (CRE)-binding sites and drive transcription. (B) Table lists unique roles of individual CRTC family members. (C) To-scale representation of the cAMP-regulated transcriptional coactivator (CRTC) protein family (according to *Mus musculus* CRTC proteins). All CRTCs contain multiple salt-inducible kinase (SIK) phosphorylation sites that mediate cytoplasmic sequestration upon 14-3-3 protein binding (black and gray). Two of these sites are conserved and function cooperatively in all CRTCs (gray: S171 and S275 in CRTC2; CBD, CREB-binding domain; TA, transactivation domain).

**Figure 2:**
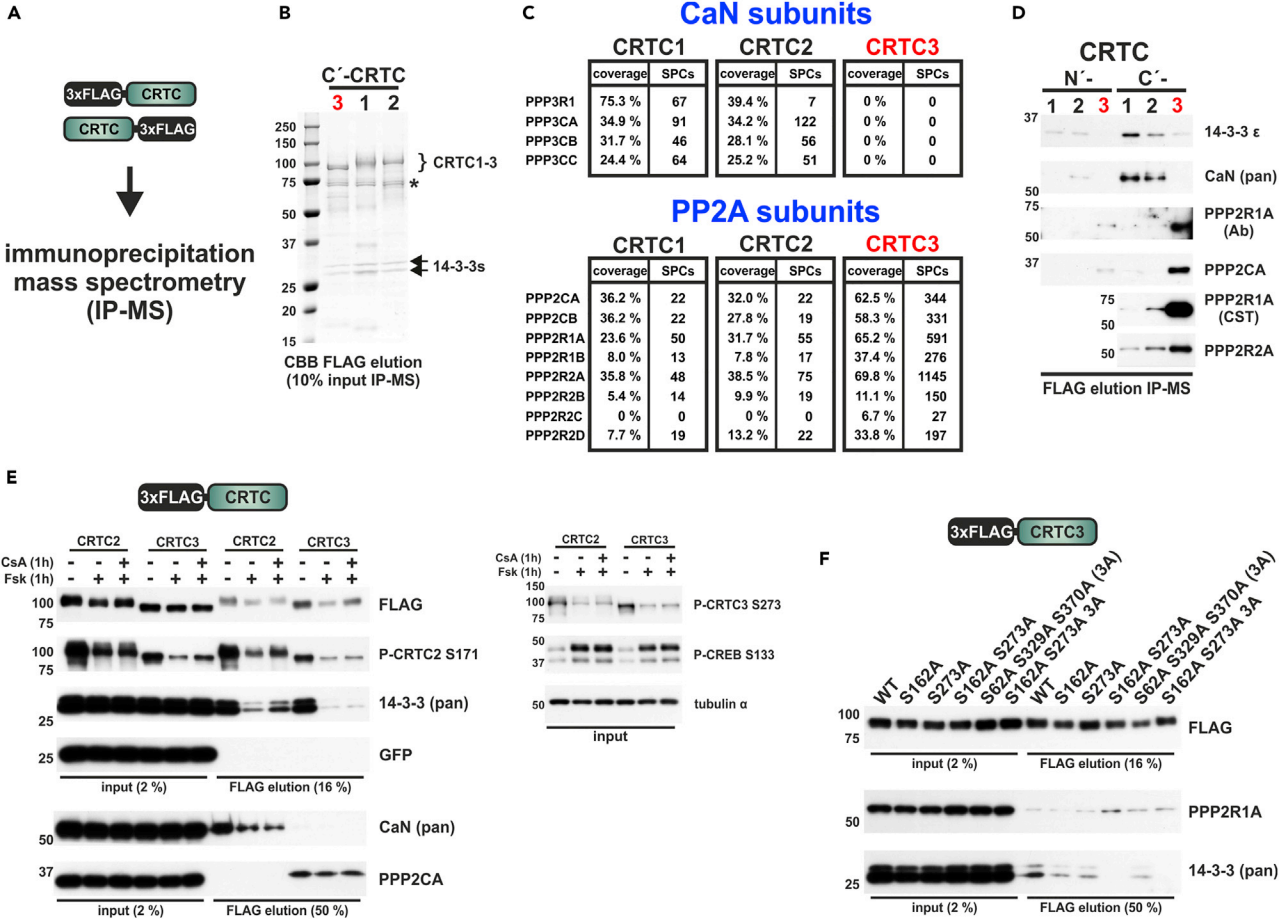
CRTC Family Members Are Regulated by Distinct Ser/Thr Protein Phosphatases (A) Overexpressed N- and C-terminally FLAG-tagged CRTC1–3 were subjected to the immunoprecipitation and mass spectrometry (IP-MS) protocol following exposure to 10 μM forskolin (Fsk) for 1 hr. (B) Coomassie brilliant blue (CBB)-stained SDS-PAGE of coIP from C-terminally FLAG-tagged CRTC1–3 subjected to MS (*, heat shock proteins). (C) Tables showing recovery of individual subunits from the Ser/Thr phosphatases calcineurin (CaN) and protein phosphatase 2 (PP2A; comparison N-terminally tagged CRTC1–3; SPCs, spectral counts). (D) Western blot analysis of the MS coIP of N- and C-terminally FLAG-tagged CRTC1–3 with endogenous 14-3-3 proteins, CaN, and PP2A (Ab, Abcam; CST, Cell Signaling Technology). (E) Western blot analysis of the coIP of FLAG-tagged CRTC2 and CRTC3 with endogenous 14-3-3 proteins, CaN, and PP2A. One hour before coIP, cells were treated with 10 μM Fsk and 100 nM cyclosporin (CsA). CRTC2 and CRTC3 were expressed from a plasmid containing the constitutive ubiquitin C promoter (UbC), which drives the expression of EGFP via an internal ribosome entry site. In HEK 293T cells the P-CREB S133 antibody recognizes two bands; the upper band corresponds to CREB1 and the lower band to ATF1 (calculated molecular weight: ∼ 37 and 29 kDa). (F) Western blot analysis of the coIP of FLAG-tagged CRTC3 14-3-3 binding site mutants with endogenous 14-3-3s and PP2A.

Although they are all regulated by cAMP and the SIKs, individual CRTC family members can perform unique roles (Figures 1A and 1B). We hypothesized that these distinct phenotypes reflect in part differences in protein-protein interactions, rather than differences in CRTC protein abundance. In previous immunoprecipitation (IP)-mass spectrometry (MS) studies, we identified two conserved SIK/14-3-3-binding sites that inhibit CRTC activity (Sonntag et al., 2017) (S171 and S275 in CRTC2; Figure 1C). Using the corresponding HEK 293T cell dataset to identify Ser/Thr phosphatases that interact with overexpressed CRTC1–3 (Figures 2A and 2B), we detected the regulatory and catalytic subunits of CaN in IPs of CRTC1 and CRTC2, but not CRTC3 (Figures 2C and 2D). Conversely, CRTC3 but not CRTC1/2 associated very strongly with PP2A holoenzymes; spectral counts for catalytic (PPP2CA/B), scaffold (PPP2R1A/B), and 55-kDa regulatory B (B55, PPP2R2A–D) subunits were more than 10-fold enriched in IPs of CRTC3 relative to CRTC1/2. Other phosphatases (PP1, PP4, and PP6) appeared to associate comparably, albeit weakly with all CRTC family members (Figure S1).

We confirmed the mutually exclusive interactions of CRTC2 with CaN and CRTC3 with PP2A by co-immunoprecipitation (coIP) (Figure 2F). Exposure to the adenylyl cyclase activator forskolin (Fsk) triggered the dephosphorylation of both CRTC2 and CRTC3 at SIK/14-3-3 sites (CRTC2/3: S171/S162 and S275/S273), thereby decreasing their association with 14-3-3 proteins. Co-treatment with the CaN inhibitor cyclosporin (CsA) (Jin and Harrison, 2002) selectively impaired the Fsk-mediated dephosphorylation of CRTC2 at S171, leading to increases in 14-3-3 binding. Fsk stimulation decreased the association of CRTC2 with CaN, but it had no effect on the association of CRTC3 with PP2A. We considered that CRTC3 phosphorylation at 14-3-3 binding sites may modulate PP2A interaction (Figure 2G). Although it eliminated 14-3-3 binding, mutation of all five SIK/14-3-3 sites (Sonntag et al., 2017) in CRTC3 had no effect on its association with PP2A. Collectively, these results indicate that the interaction of CRTC3 with the B55 PP2A holoenzyme is independent of SIK-mediated phosphorylation.

### Identification of a Conserved PP2A-Binding Region in CRTC3

**Figure 3:**
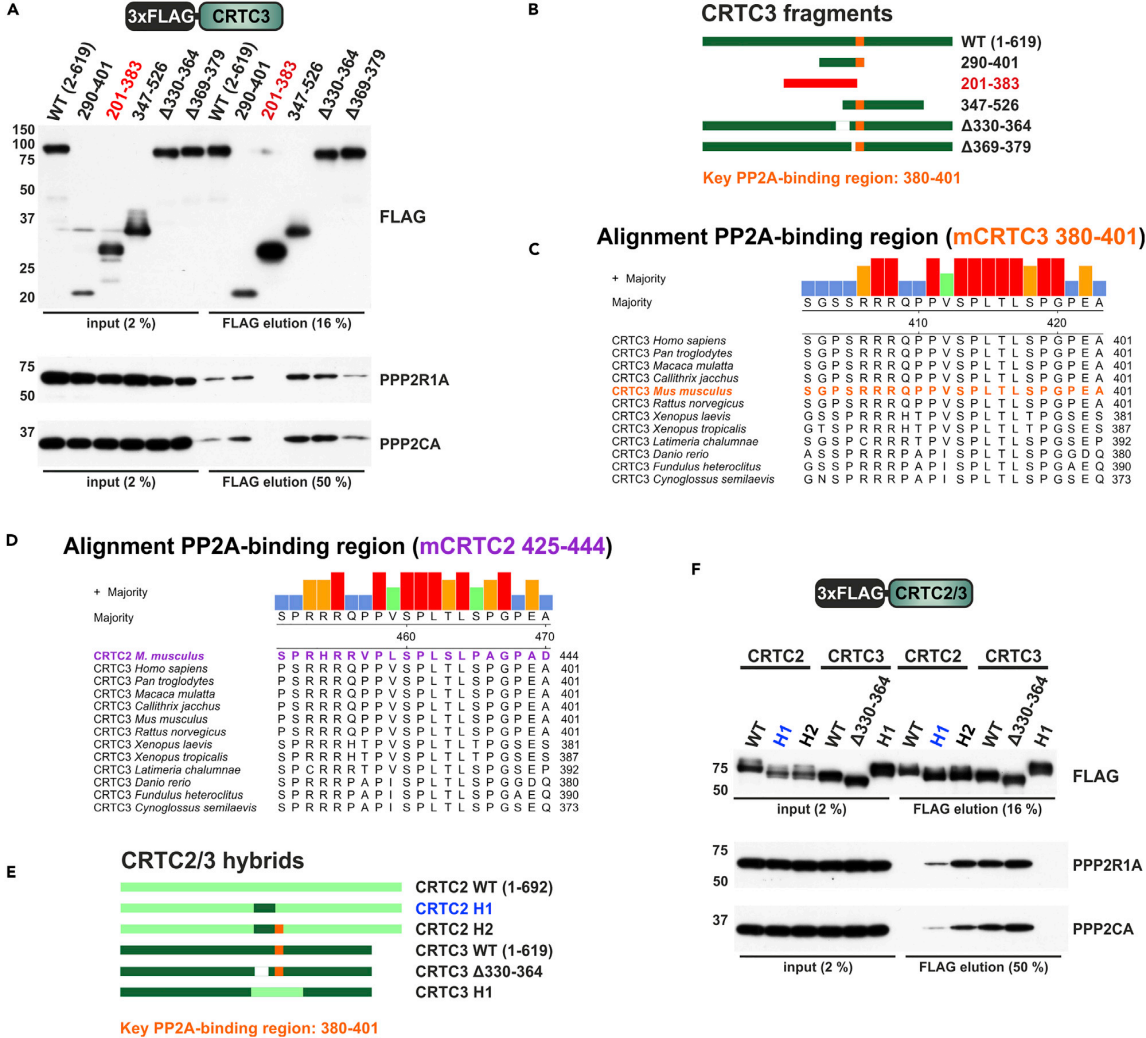
Characterization of the PP2A-Binding Region (PBR) in CRTC3 (A) Western blot analysis showing recovery of endogenous PP2A from IPs of FLAG-tagged CRTC3 mutants. The only fragment that abolished PP2A binding is highlighted in red (201–383). (B) To-scale representation of CRTC3 protein fragments assayed for interaction with PP2A. In orange the PBR of CRTC3 (*Mus musculus* amino acids 380–401). (C) CRTC3 sequence alignment of the PBR from multiple vertebrate species (CRTC3 sequences were obtained via UniProt; The UniProt Consortium, 2017). (D) Sequence alignment of the PBR from vertebrate CRTC3 proteins compared with *M. musculus* CRTC2 425–444 (in purple). (E and F) (E) To-scale representation of the CRTC2 (light green) and CRTC3 (dark green) hybrid proteins assayed for PP2A binding. Hybrid proteins: CRTC2 H1 [mCRTC2(328–421) replaced with mCRTC3(326–379), in blue], CRTC2 H2 [mCRTC2(328–449) replaced with mCRTC3(326–402)], and CRTC3 H1 [mCRTC3(322–407) replaced with mCRTC2(324–454)]. (F) Western blot showing recovery of endogenous PP2A from IPs of FLAG-tagged CRTC2/3 mutants.

To characterize the PP2A-binding region (PBR) in CRTC3, we generated serial N- and C-terminal truncations of CRTC3 and analyzed these for PP2A association by coIP (Figure S2). This analysis uncovered a 20-amino acid core PBR (amino acids 380–401) located within the central regulatory domain of CRTC3 (Figures 3A and 3B). Pointing to an important function for this region, the PBR amino acid sequence is identical between humans and mice (Figure 3C).

Although CRTC2 does not bind PP2A, it contains a PBR-related sequence at 425–444 (Figure 3D). We generated CRTC2/3 hybrids to test whether sequences flanking this core region modulate the PP2A association (Figures 3E and 3F). Similar to the PP2A interaction patterns of truncated CRTC3 protein fragments, insertion of mCRTC3 326–402 promoted binding of CRTC2 to PP2A (CRTC2 H2), whereas insertion of the corresponding region of CRTC2 (mCRTC2 324–454) into CRTC3 (CRTC3 H1) abolished PP2A binding. However, insertion of CRTC3 sequences flanking the PBR into CRTC2 (mCRTC3 326–379; CRTC2 H1) increased PP2A binding to a level that was intermediate between CRTC2 and CRTC3. These findings indicate that although mCRTC3 380–401 is absolutely required for the strong interaction with PP2A, regions outside the PBR also contribute to complex formation.

### Phosphorylation of the PBR Increases PP2A Binding

**Figure 4:**
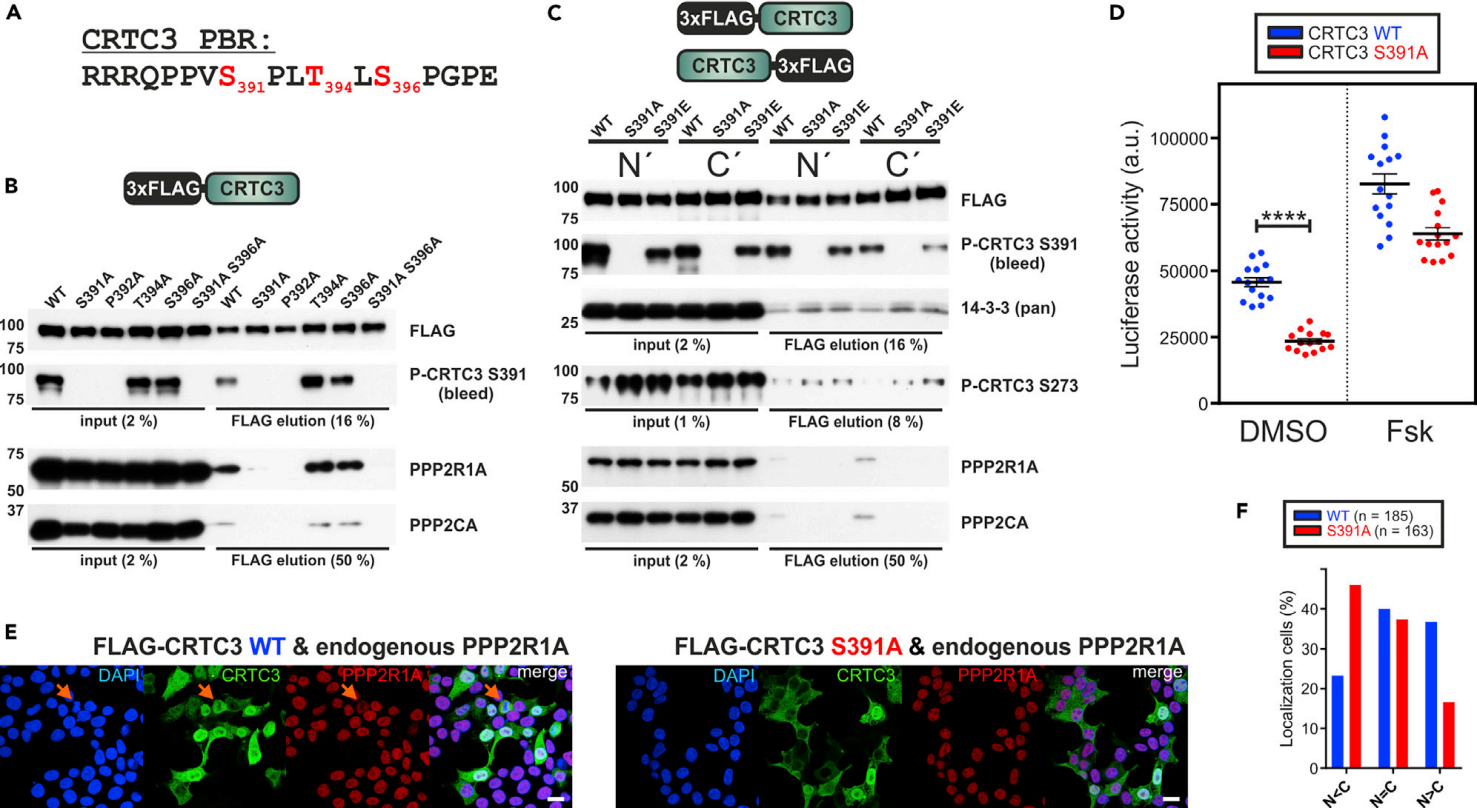
Phosphorylation of CRTC3 at S391 Promotes PP2A Association and CRTC3 Activation (A) Primary sequence of the PP2A-binding region (PBR) in CRTC3 (*H. sapiens* and *Mus musculus* numbering is identical). Phosphorylated residues in red (Hornbeck et al., 2015). (B) Western blot analysis showing recovery of endogenous PP2A from IPs of FLAG-tagged CRTC3 mutants. (C) Western blot analysis of endogenous PP2A and 14-3-3s recovered from IPs of FLAG-tagged CRTC3 S391 mutants. (D–F) (D) EVX-Luc reporter assay measuring the effect of overexpressed CRTC3 wild-type (WT) and S391A mutant upon CREB-dependent gene expression. EVX-Luc activity was measured after 4 hr of DMSO/Fsk treatment (10 μM) (n = 15, ±SEM; ****p < 0.0001). (E) Immunofluorescence of HEK 293T cells transfected with FLAG-tagged CRTC3 WT and S391A. Cells were stained for FLAG epitope, PPP2R1A, and counterstained with DAPI. (Orange arrows depict cells in mitosis; scale bar, 20 μm.) (F) Graph depicting the relative subcellular localization of CRTC3 WT and S391 mutant (predominantly cytoplasmic [N < C], comparable [N=C], and predominantly nuclear distribution [N > C]).

The CRTC3 PBR contains three conserved Ser/Thr residues that appear to undergo phosphorylation (Hornbeck et al., 2015) (Figure 4A). We confirmed the phosphorylation of CRTC3 at S391 and S396 by MS analysis (Figure S3). To assess the potential effects of these modifications within the PBR on the CRTC3-PP2A interaction, we performed mutagenesis studies (Figure 4B). Although alanine substitutions at T394 and S396 had no effect on PP2A binding, mutation of either S391 or the flanking P392 to alanine abolished this interaction. To monitor phosphorylation at the critical S391 site, we generated a phospho-specific antiserum (P-CRTC3 S391). Western blot analysis revealed robust S391 phosphorylation following overexpression of CRTC3 wild-type (WT) and T394A or S396A mutant proteins in HEK 293T cells.

To determine the importance of S391 phosphorylation for the CRTC3-PP2A interaction, we tested whether substitution of this phosphoacceptor with alanine or a phosphomimetic (Glu) alters complex formation (Figure 4C). In contrast to WT CRTC3, neither S391A nor S391E mutant CRTC3 protein associated detectably with PP2A. Correspondingly, phospho-S391-defective CRTC3 proteins were phosphorylated to a greater extent at 14-3-3 binding sites (P-CRTC3 S273), and as a result, the association of S391A/E mutants with 14-3-3 proteins was stronger compared with WT.

In a transient CRE luciferase-based assay for the quantification of cAMP-dependent gene expression (Conkright et al., 2003), the phosphorylation-defective S391A mutant CRTC3 exhibited ∼2-fold lower basal transcriptional activity compared with WT CRTC3 (Figure 4D). WT and S391A CRTC3 CRE activities were more similar following exposure to Fsk, however, suggesting that S391 modulates CRTC3 activity in a cAMP-independent manner. We performed immunofluorescence studies to determine the subcellular localization of the S391A mutant (Figures 4E and 4F). In contrast to the predominantly nuclear WT CRTC3, S391A mutant protein was primarily localized in the cytoplasm under basal conditions. In keeping with its effects on CRE reporter activity, Fsk promoted the nuclear translocation of CRTC3 WT and S391A comparably (Figure S4).

PP2A has multifaceted roles in the cell cycle and is thought to function as a tumor suppressor (Haesen et al., 2014, Wlodarchak and Xing, 2016). Various cellular proteins can inhibit PP2A activity, including the phosphorylation-dependent regulators of mitosis ENSA, ARPP19, and Bod1 (Gharbi-Ayachi et al., 2010, Porter et al., 2013). In contrast to CRTC3 (Song et al., 2010), these proteins are small (12–20 kDa) (The UniProt Consortium, 2017) and knockdown or overexpression of any one regulator typically leads to growth arrest and embryonic lethality (Dupre et al., 2014, Matthews and Evans, 2014). Arguing against a role for CRTC3 as a PP2A inhibitor, cellular PP2A activity and proliferation appeared comparable between cells expressing WT or phosphorylation-defective (S391A) CRTC3 in HEK 293T cells (Figure S5). These results support the notion that S391 phosphorylation selectively increases CRTC3 activity by promoting its association with the PP2A holoenzyme, which in turn dephosphorylates CRTC3 at remote SIK/14-3-3 sites, leading to CRTC3 nuclear translocation and CREB target gene activation.

### MAPKs and CDKs Promote PBR Phosphorylation at S391

**Figure 5:**
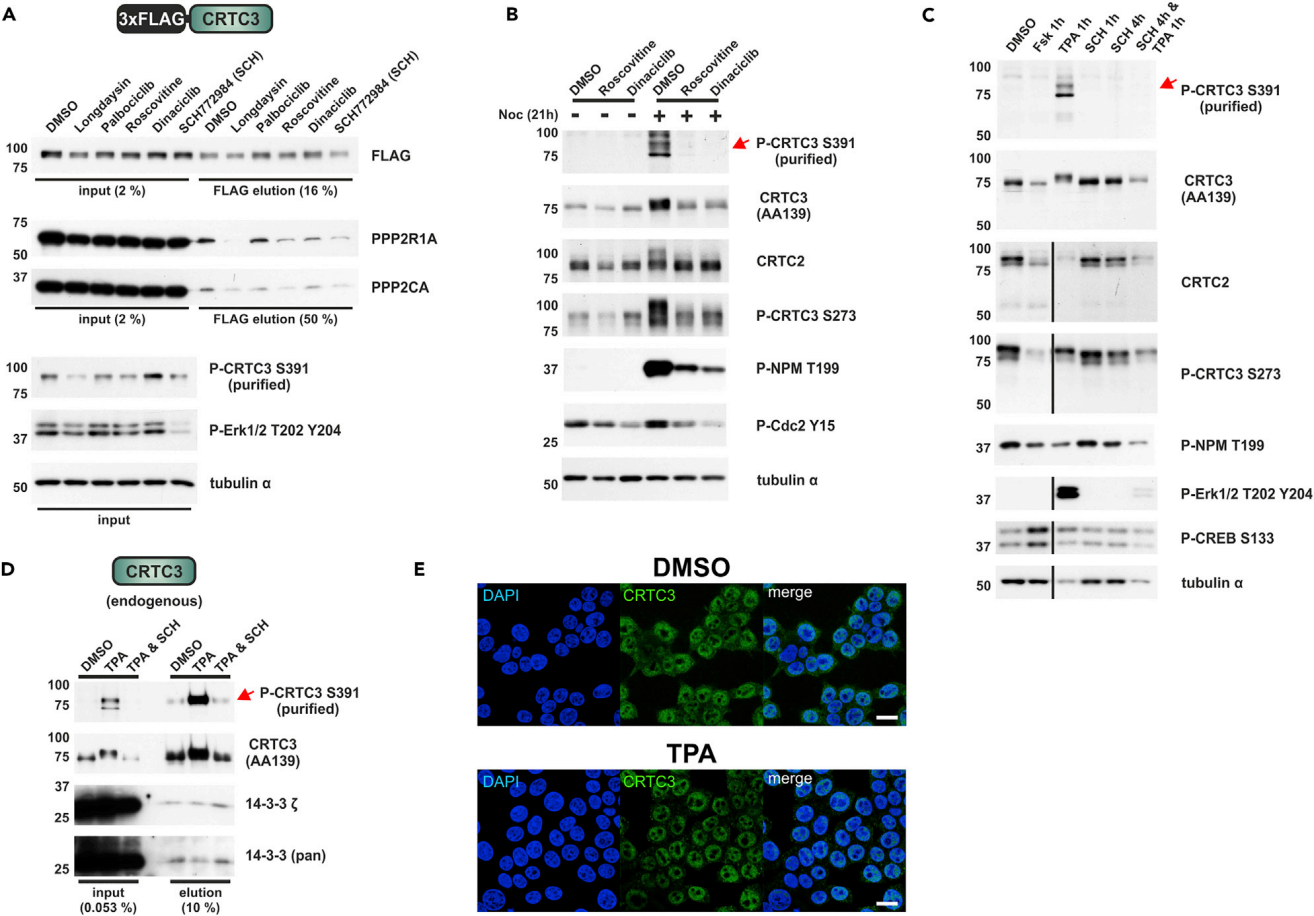
MAPKs and CDKs Promote S391 Phosphorylation and Activation of CRTC3 (A) Western blot analysis of endogenous PP2A recovered from IPs of FLAG-tagged CRTC3. Four hours before the IP, HEK 293T cells were treated with small molecule kinase inhibitors: 20 μM longdaysin (IC_50_: CK1α/δ = 6–9 μM, ERK2 = 52 μM) (Hirota et al., 2010), 1 μM palbociclib (IC_50_: CDK4/6 = 0.009–0.015 μM) (Fry et al., 2004), 20 μM roscovitine (IC_50_: CDK1/2/5 = 0.2–0.8 μM, ERK1/2 = 15–30 μM) (Meijer et al., 1997), 100 nM dinaciclib (IC_50_: CDK1/2/5 = 0.001–0.003 μM) (Parry et al., 2010), and 100 nM SCH772984 (IC_50_: ERK1/2 = 0.004/0.001 μM) (Morris et al., 2013). (B) Western blot analysis showing the effects of 21-hr exposure to nocodazole (noc, 0.1 μg/mL) on S391 phosphorylation of endogenous CRTC3. Four hours before cell lysis, HEK 293T cells were treated with roscovitine (20 μM) and dinaciclib (200 nM). In HEK 293T cells the P-CRTC3 S273 antibody recognizes two bands, the upper band being CRTC2 and the lower band CRTC3 (calculated molecular weight: ∼ 73 and 67 kDa). (C) Western blot analysis showing the effects of 1-hr TPA (200 nM) and Fsk (10 μM) treatment on S391 phosphorylation of endogenous CRTC3. In addition, HEK 293T cells were (co-)treated with SCH772984 (SCH; 100 nM) for the indicated time before cell lysis. (D) Western blot analysis of the coIP of endogenous CRTC3 with 14-3-3 proteins by using the hCRTC3(414-432) antibody. Before the IP, HEK 293T cells were (co-)treated with TPA (200 nM, 1 hr) and SCH772984 (SCH; 200 nM, 2 hr). (E) Immunofluorescence of HEK 293T cells following 30 minutes of DMSO and TPA (200 nM) stimulation. Cells were stained for endogenous CRTC3 with the hCRTC3(414–432) antibody and counterstained with DAPI (scale bar, 20 μm).

Realizing that S391 phosphorylation of CRTC3 increases its interaction with PP2A, we set out to identify relevant protein kinases that mediate the phosphorylation of this site. Based on the importance of a flanking proline at the +1 position relative to S391, we addressed the potential involvement of proline-directed kinases in this process by pharmacological inhibition (Figure 5A). Exposure to the cyclin-dependent kinase (CDK1/2/5) inhibitor roscovitine (IC_50_: CDK1/2/5 = 0.2–0.8 μM, ERK1/2 = 15–30 μM) (Meijer et al., 1997) and the ERK1/2 inhibitor SCH772984 (IC_50_: ERK1/2 = 0.004/0.001 μM) (Morris et al., 2013) reduced both CRTC3 S391 phosphorylation and PP2A binding following CRTC3 overexpression in HEK 293T cells. CDKs (Holt et al., 2009) and mitogen-activated protein kinases (MAPKs) such as ERK1/2 (Carlson et al., 2011) are proline-directed Ser/Thr kinases that appear capable of phosphorylating CRTC3 at S391. The CK1α/δ inhibitor longdaysin (IC_50_: CK1α/δ = 6–9 μM, ERK2 = 52 μM) (Hirota et al., 2010) also inhibited S391 phosphorylation, with the PBR (S_391_PLT_394_) containing a casein kinase 1 (CK1) consensus motif pS/Txx(x)S/T (underlined = CK1 phosphorylated residue) (Knippschild et al., 2014). In contrast to its effects on WT CRTC3, longdaysin did not impair PP2A binding in the context of the T394A mutant (Figure S6), indicating that S391 and T394 phosphorylation are required for full binding of PP2A in cells overexpressing WT CRTC3.

To evaluate the potential role of CDKs in modulating endogenous CRTC3 S391 phosphorylation, we arrested HEK 293T cells in the G2/M phase with nocodazole (Figure 5B). Although undetectable in the basal state, phosphorylations of CRTC3 at S391 and nucleophosmin (NPM) at T199, a known CDK target (Tokuyama et al., 2001), were both upregulated following nocodazole arrest. Consistent with these effects, short-term CDK inhibition with roscovitine and dinaciclib (IC_50_: CDK1/2/5 = 0.001–0.003 μM) (Parry et al., 2010) blocked the phosphorylation of both proteins.

To test the effect of ERK and cAMP signaling on endogenous S391 phosphorylation of CRTC3, we stimulated HEK 293T cells with either 12-O-tetradecanoylphorbol-13-acetate (TPA) (Schonwasser et al., 1998) or Fsk (Figure 5C). In contrast to Fsk, which promoted the dephosphorylation of CRTC2 and CRTC3 at 14-3-3 sites (P-CRTC3 S273: top CRTC2 and bottom CRTC3), exposure to TPA upregulated ERK activity (P-ERK T202 Y204) and induced CRTC3 phosphorylation at S391. These effects were suppressed by pretreatment with the ERK1/2 inhibitor SCH772984.

Following exposure to TPA or nocodazole, we detected two immunoreactive bands using the S391 phospho-specific antiserum. Both bands correspond to endogenous CRTC3, as revealed in knockdown studies using CRTC3-specific short hairpin RNA (shRNAs) (Figure S7). In previous studies with HEK 293T cells, we found that TPA does not activate the predominant CRTC family member CRTC2 (Ravnskjaer et al., 2007). By contrast, exposure to TPA decreased 14-3-3 protein binding to CRTC3, thereby enhancing its nuclear localization (Figures 5D and 5E). Taken together, these results indicate that MAPKs and CDKs both regulate the S391 phosphorylation and activation of endogenous CRTC3.

### PBR Phosphorylation Increases CRTC3 Activity

**Figure 6:**
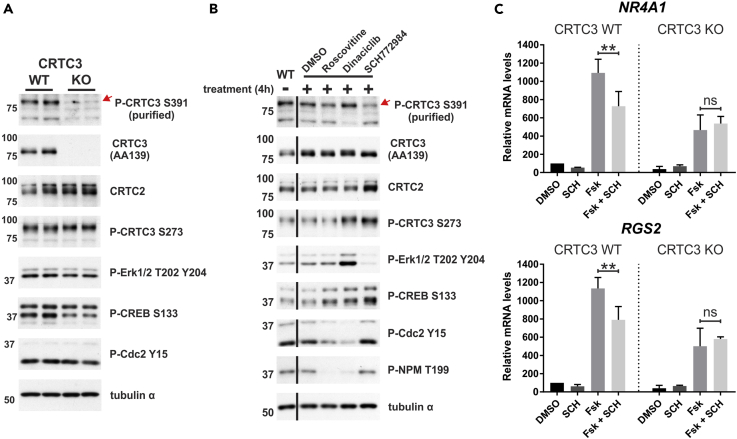
ERK-Mediated Activation of CRTC3 in Undifferentiated bSVFs (A) Western blot analysis of primary brown adipose stromal vascular fraction (bSVF) cells generated from wild-type C57BL/6 (WT) and CRTC3 knockout mice (KO). (B) Western blot analysis showing the effects of 4-hr treatment with roscovitine (20 μM), dinaciclib (200 nM), and SCH772984 (200 nM) on phospho-S391 CRTC3 levels in bSVF WT and KO cells. (C) *NR4A1* and *RGS2* mRNA levels of bSVF CRTC3 WT and KO cells following (co-)treatment with SCH772984 (SCH; 200 nM, 4 hr) and Fsk (10 μM, 1 hr). Values represent data from three independent experiments normalized to *RPL32* and relative to CRTC3 WT DMSO. (*n* = 3, ±SD; ns, not statistically significant, **p < 0.01)

Based on the importance of CRTC3 for adipose function (Song et al., 2010, Yoon et al., 2018), we examined CRTC3 S391 phosphorylation in brown-adipose-tissue-derived stromal vascular fraction (bSVF) cells, which are enriched in preadipocytes (Han et al., 2015) (Figure 6A). In the basal state, S391-phosphorylated CRTC3 was detected in WT, but not in CRTC3 knockout (KO) bSVFs. Having seen their effects on CRTC3 S391 phosphorylation in HEK 293T cells, we tested CDK and ERK inhibitors in bSVFs (Figure 6B). Although both roscovitine and dinaciclib inhibited CDK activity as revealed by the corresponding reduction in NPM T199 phosphorylation, the ERK1/2 inhibitor SCH772984 was most effective in reducing CRTC3 S391 phosphorylation. We evaluated the importance of ERK signaling for CREB target gene expression by exposing bSVFs to SCH772984 (Figure 6C). SCH772984 treatment partially blocked the Fsk-mediated induction of canonical CREB target genes *NR4A1* and regulator of G-protein signaling-2 (*RGS2*) (Ravnskjaer et al., 2007, Song et al., 2010). The inhibitory effects of SCH772984 appear to be CRTC3 dependent, because they were absent in CRTC3 KO bSVFs. Taken together, these results demonstrate that in bSVFs ERK activity functions cooperatively with cAMP/PKA signals to promote CREB target gene expression through its effects on CRTC3.

## Discussion

**Figure 7:**
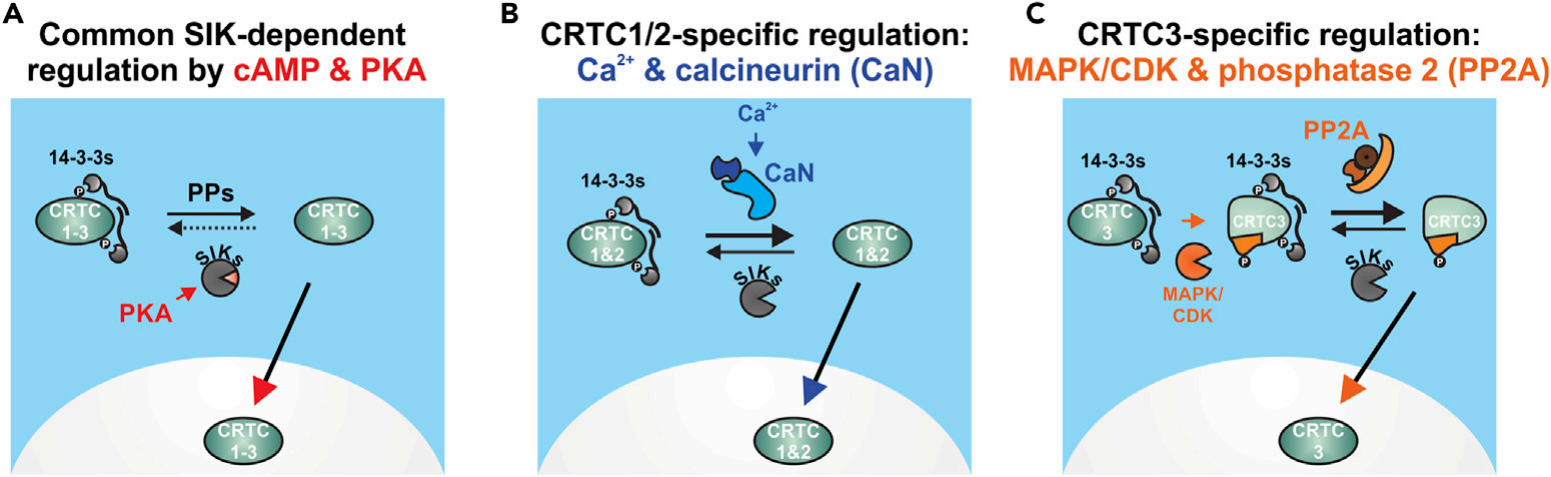
Differential Regulation of CRTC Family Members by Calcineurin and B55 PP2A (A) Scheme depicting the cAMP-dependent regulation of CRTC1–3. In the basal state, SIK-mediated phosphorylation sequesters the CRTCs in the cytoplasm by inducing 14-3-3 interactions. Upon cAMP stimulation, PKA-mediated phosphorylation inactivates the SIKs. CRTCs are dephosphorylated by protein phosphatases (PPs), lose 14-3-3 binding, and translocate to the nucleus. (B) Scheme depicting the regulation of CRTC1 and CRTC2 by calcineurin. Upon Ca^2+^ stimulation, increased calcineurin activity dephosphorylates 14-3-3 binding sites, leading to nuclear translocation of CRTC1/2. (C) Scheme depicting the regulation of CRTC3 by PP2A. Phosphorylation by MAPKs or CDKs at S391 within the PBR (amino acids 380–401) of CRTC3 enables B55 PP2A holoenzyme recruitment and subsequent dephosphorylation of CRTC3 at 14-3-3 binding sites.

The second messenger cAMP promotes cellular gene expression via the PKA-mediated phosphorylation and inhibition of the SIKs, leading to CRTC nuclear translocation upon dephosphorylation and release from 14-3-3 proteins (Figure 7A). Here we show that the association of CRTC members with different protein phosphatases provides distinct stimulus-dependent mechanisms for the induction of CREB target genes (Figures 7B and 7C).

The calcium-responsive phosphatase CaN interacts with substrates that contain PxIxIT motifs (Li et al., 2011), including vertebrate CRTC1 and CRTC2, but not CRTC3 (mCRTC1: P_219_GINIF, P_555_NIILT and mCRTC2: P_249_GINIF, P_614_NIILT). Although PxIxIT motifs regulate the substrate affinity of CaN (Li et al., 2007, Roy et al., 2007), its phosphatase activity is dependent upon Ca^2+^/calmodulin (Perrino et al., 1995). As a result, the Ca^2+^/CaN -dependent regulation is limited to CRTC1 and CRTC2 (Altarejos et al., 2008, Kovacs et al., 2007, Screaton et al., 2004, Bittinger et al., 2004, Ch'ng et al., 2012).

By contrast, CRTC3 binds to the B55 PP2A holoenzyme following phosphorylation of CRTC3 at S391 by MAPK/CDKs. Within the PBR (mCRTC3 PBR: 380–401) the S391 phosphorylation site (PVS_391_P) forms part of an optimal ERK1/2 motif (Px[S/T]P) (Carlson et al., 2011). The PBR also contains an MAPK docking motif (D domain), with characteristic basic and alternating hydrophobic residues (RRRQPPVS_391_PLTL) (Remenyi et al., 2005). Stimuli that upregulate ERK activity (epidermal growth factor and angiotensin II), also promote the phosphorylation of CRTC3 at S391/S396 (Olsen et al., 2006, Christensen et al., 2010). Superimposed on effects of ERKs, CDKs also appear to phosphorylate CRTC3 at S391 in growth-arrested cells (Olsen et al., 2010, Franz-Wachtel et al., 2012). Indeed, CRTC3 was also identified in a study of mitotic phosphorylation substrates within the chromosomal passenger complex (Yang et al., 2007), which controls cell division together with the CDKs (Carmena et al., 2012). Consistent with the importance of PP2A-mediated activation of CRTC3, in primary calvarial osteoblasts the parathyroid-hormone-induced nuclear translocation of CRTCs was blocked by the PP2A/PP1 inhibitor okadaic acid in case of CRTC3, but not for CRTC2 (Ricarte et al., 2018).

Although the regulatory subunits of PP2A appear important in distinguishing between different substrates, relevant substrate recognition motifs for the PP2A holoenzyme have not been reported until recently (Haesen et al., 2014, Wlodarchak and Xing, 2016). The B55 PP2A holoenzyme has been found to recognize substrates containing a CDK1 phosphorylation site flanked on both sides by a polybasic region (Cundell et al., 2016); a short LxxIxE motif appears to mediate interaction with the B56 PP2A holoenzyme (Hertz et al., 2016). The PBR of CRTC3 includes an N-terminal Arg triplet but lacks any basic C-terminal sequences (mCRTC3: RRRQPPVS_391_PLTLSPGPE). Only B55 PP2A subunits were recovered from IPs of all CRTCs, which contain a common sequence inside their PBR-related region consisting of two hydrophobic residues flanking an SP motif (mCRTC1/2/3: PLS_332_PITQ/PLS_434_PLSL/PVS_391_PLTL). Although it lacks an essential glutamate at position 6, this common PBR-like motif in CRTC family members bears more similarity with the LxxIxE motif described for the B56 PP2A holoenzyme. Similar to our findings, phosphorylation of residues within the LxxIxE motif also increases B56 PP2A affinity, leading to the dephosphorylation of distal residues in relevant substrates such as Repo-Man (LSPIPE), BubR1 (LSPIIE), and RacGAP1 (LSTIDE; underlined = phosphorylated residues) (Hertz et al., 2016, Qian et al., 2013, Kruse et al., 2013). Future biochemical studies should reveal the extent to which the PBR we identified represents a distinct binding motif for the B55 PP2A holoenzyme.

The second messenger cAMP is traditionally thought to inhibit cell division, but in a subset of tissues, cAMP appears to cooperate with MAPK/ERK signals in promoting proliferation and differentiation (Dumaz and Marais, 2005). This cross talk is noteworthy in adipose tissue wherein β-adrenergic signaling triggers the activation of both cAMP and MAPK signaling pathways (Collins, 2011), often in combination with growth factors such as insulin/insulin growth factor-1 (Kajimura et al., 2015, Tang and Lane, 2012). As loss of CRTC3 expression increases brown adipocyte proliferation and differentiation and protects against high-fat-diet-induced adipose expansion (Song et al., 2010, Yoon et al., 2018), future studies should address the specific roles of S391 phosphorylation in this context. This cooperative effect of cAMP and MAPK signals on CRTC3 activity has also been reported in regulatory macrophages where lipopolysaccharides (LPS) and prostaglandin E2 (PGE2) promote expression of the anti-inflammatory interleukin 10 (Clark et al., 2012, MacKenzie et al., 2013). Based on the ability of PGE2 to stimulate the cAMP pathway and LPS to activate multiple MAPK family members (Symons et al., 2006), our results suggest that these CRTC3-specific effects proceed via the recruitment of B55 PP2A.

### Limitations of the Study

We used HEK 293T cells to characterize CRTC3-PP2A complex formation and its regulation by MAPK- and CDK-mediated phosphorylation. Although this approach was valuable in characterizing these molecular components and their downstream effects on CRTC3 activation, the relevant extracellular signals that act upstream to trigger the interaction of CRTC3 with PP2A remain relatively unknown. Our studies with brown pre-adipocytes support a potential role for ERK in mediating effects of β-adrenergic signals on recruitment of PP2A and on the subsequent activation of CRTC3. Future studies with phospho (S391)-specific antiserum should reveal the physiological contexts in which this site is phosphorylated, and mutant mice expressing phospho-S391 defective CRTC3 should provide insight into the physiological importance of this modification.

## Methods

All methods can be found in the accompanying Transparent Methods supplemental file.

## Supplemental Information

Document S1. Transparent Methods and Figures S1–S7
