# Supplementary material for: Mitogenic Signals Stimulate the CREB Coactivator CRTC3 through PP2A Recruitment
Source: iScience. 2018 Dec 19;11:134–45. doi: 10.1016/j.isci.2018.12.012 (PMC6317279; doi:10.1016/j.isci.2018.12.012)
Supplement: Document S1. Transparent Methods and Figures S1–S7 [file mmc1.pdf]

ISCI, Volume *11*

Supplemental Information

Mitogenic Signals Stimulate the CREB Coactivator CRTC3 through PP2A Recruitment

Tim Sonntag, Jelena Ostojić, Joan M. Vaughan, James J. Moresco, Young-Sil Yoon, John R. Yates III, and Marc Montminy

Supplemental Figures

Fig. S1

PP1 subunits

	CRTC1		CRTC2		CRTC3	
	coverage	SPCs	coverage	SPCs	coverage	SPCs
PPP1CA	11.8 %	16	9.7 %	16	40.6 %	20
PPP1CB	13.5 %	14	9.8 %	16	37.6 %	18
PPP1CC	8.7 %	7	9.9 %	16	27.9 %	17
PPP1R10	2.3 %	2	5.7 %	3	3.8 %	5

PP4 subunits

	CRTC1		CRTC2		CRTC3	
	coverage	SPCs	coverage	SPCs	coverage	SPCs
PPP4C	10.7 %	4	20.2 %	7	43.3 %	56
PPP4R2	0 %	0	2.6 %	2	7.0 %	3
PPP4R3A	6.2 %	5	3.8 %	3	14.4 %	12

PP6 subunits

	CRTC1		CRTC2		CRTC3	
	coverage	SPCs	coverage	SPCs	coverage	SPCs
PPP6C	21.3 %	9	11.5 %	3	41.6 %	15
PPP6R1	0 %	0	0 %	0	3.0 %	2
PPP6R3	4.0 %	8	8.0 %	13	9.8 %	17

Fig. S1. Interaction of CRTC1-3 with protein phosphatases 1, 4, and 6. Related to Fig. 2.

Tables show the IP-MS recovery of protein phosphatases (PP) 1, 4, and 6 subunits (comparing N-terminally tagged CRTC1-3; SPCs = spectral counts).

Fig. S2

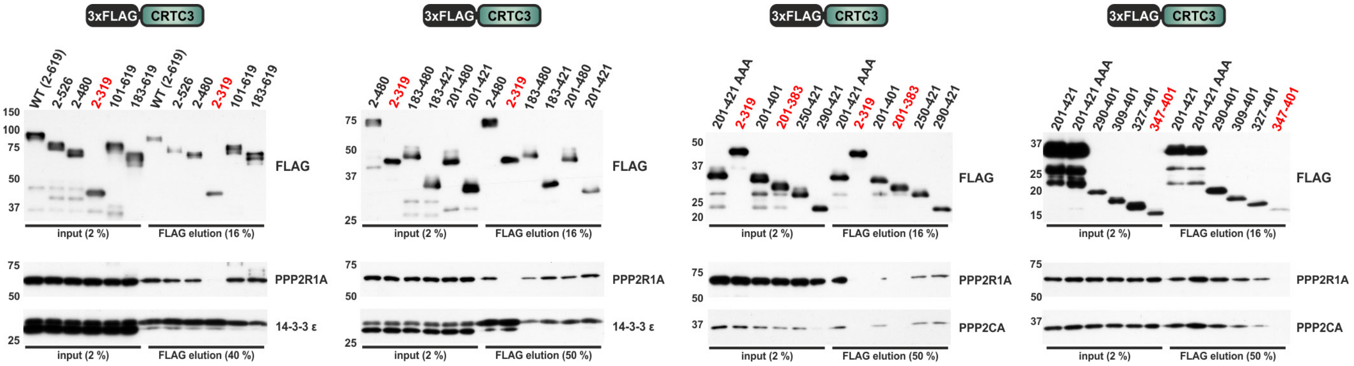

Fig. S2. Characterization of the PP2A-binding region (PBR) in CRTC3. Related to Fig. 3.

Western blot analysis of Co-IPs of FLAG-tagged CRTC3 truncation mutants with endogenous PP2A and 14-3-3 proteins. Mutants that abolished PP2A interaction are highlighted in red (201-421 AAA = mCRTC3 201-421 S273A S329A S370A).

Fig. S3

A

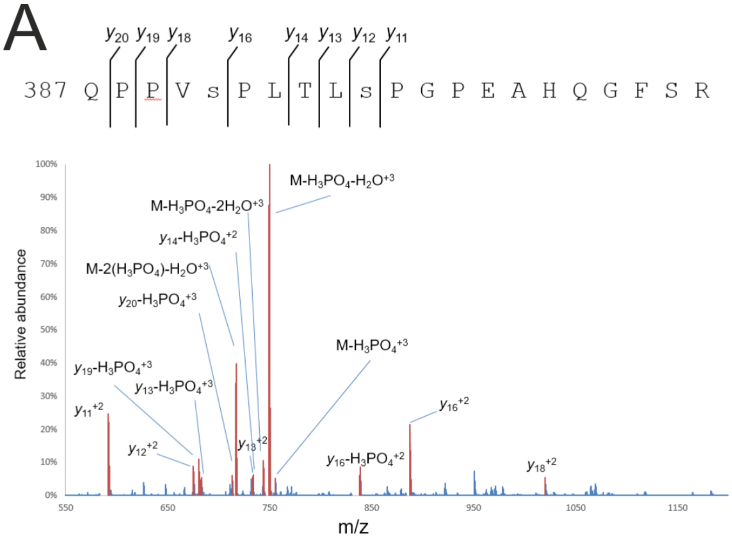

B

Peak	M/Z	Label
1	591.7890	y_{11}^{+2}
2	675.2883	y_{12}^{+2}
3	680.3337	y_{19}-$H_3PO_4^{+3}$
4	682.8434	y_{13}-$H_3PO_4^{+3}$
5	713.3282	y_{20}-$H_3PO_4^{+3}$
6	716.7068	M-2(H_3PO_4)-H_2O^{+3}
7	731.8336	y_{13}^{+2}
8	733.8691	y_{14}-$H_3PO_4^{+2}$
9	743.6959	M-H_3PO_4-2H_2O^{+3}
10	749.3690	M-H_3PO_4-H_2O^{+3}
11	755.7042	M-$H_3PO_4^{+3}$
12	838.4369	y_{16}-$H_3PO_4^{+2}$
13	887.4260	y_{16}^{+2}
14	1020.4977	y_{18}^{+2}

Fig. S3. Phosphorylation of S391 and S396 within the PP2A-binding region (PBR) of CRTC3. Related to Fig. 4.

A) Detection of MS spectra (387-407) derived from the PBR with corresponding peaks labeled (in B)). Phosphorylation was detected on amino acid s391 (Ascore = 14.89) and s396 (Ascore = 7.53; for Ascore see (Beausoleil et al., 2006)).

Fig. S4

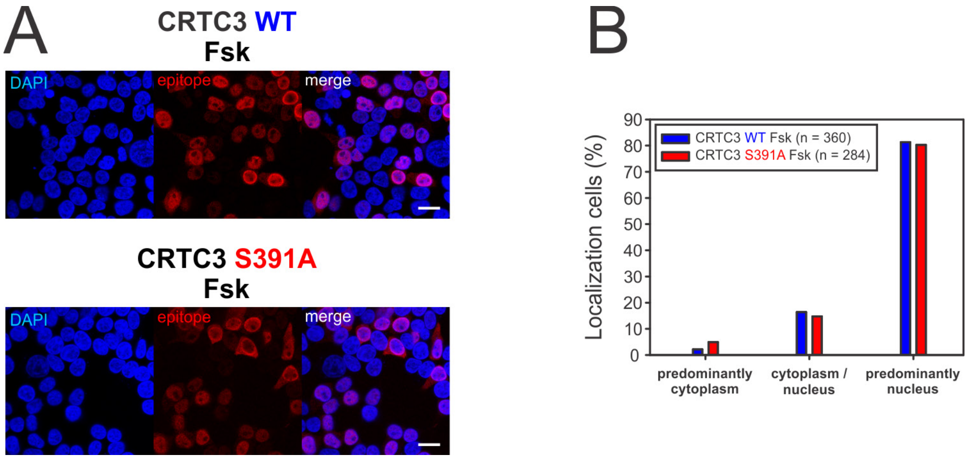

Fig. S4. Localization of CRTC3 WT and S391A mutant upon Forskolin treatment. Related to Fig. 4.

A) Immunofluorescence of HEK 293T cells transfected with FLAG-tagged CRTC3 WT and S391A. Cells were stained for FLAG epitope and counterstained with DAPI. (Fsk treatment for 30 min; scale bar indicates 20 μm) B) Graph shows relative subcellular localization of CRTC3 WT and S391A mutant.

Fig. S5

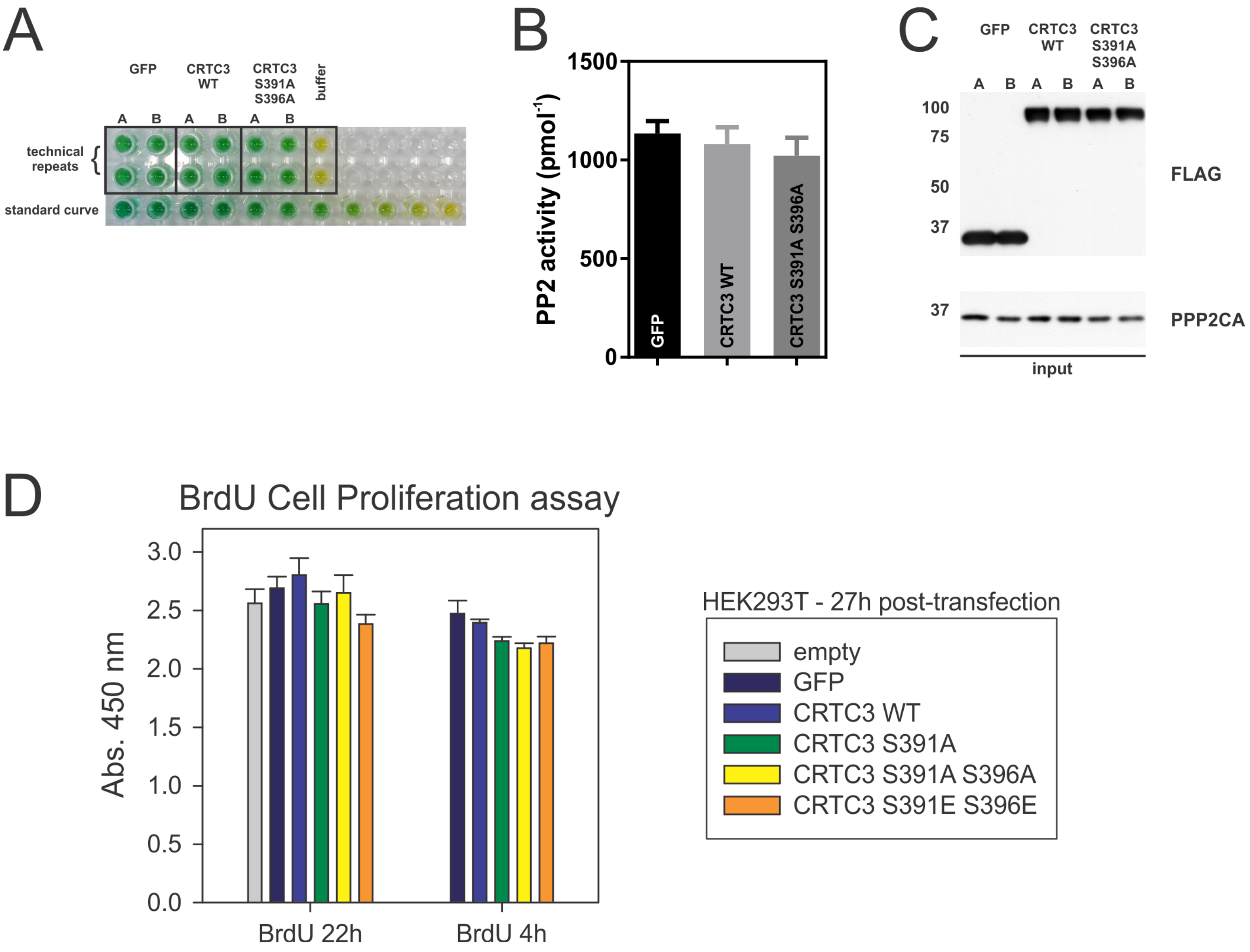

Fig. S5. Effect of CRTC3 overexpression on cellular PP2A activity and cell proliferation. Related to Fig. 4. A-C) The activity of immunoprecipitated catalytic PP2A subunit (PPP2CA) was quantified by malachite green and KRpTIRR phosphopeptide (PP2A Immunoprecipitation Phosphatase Assay Kit, Upstate). Prior to PP2A activity measurement FLAG-tagged GFP, CRTC3 and CRTC3 S391A S396A were overexpressed in HEK 293T cells for 48 h. A) Picture of the 96 well plate after the colorimetric PP2A assay. (A/B indicates independent transfections). B) Corresponding PP2A activity upon normalization to the standard curve. (n = 4, ± SD). C) Corresponding Western blot analysis of the cell lysate used for the PP2A activity assay. D) BrdU Cell Proliferation ELISA Kit (Abcam) measuring the effects of transient-overexpression of GFP, CRTC3, and CRTC3

mutants on HEK 293T proliferation. BrdU treatment occurred either for 22 h (5 h post-transfection) or for 4 h (23 h post-transfection) prior to the colorimetric assay. (*n* = 5, ± SEM).

Fig. S6

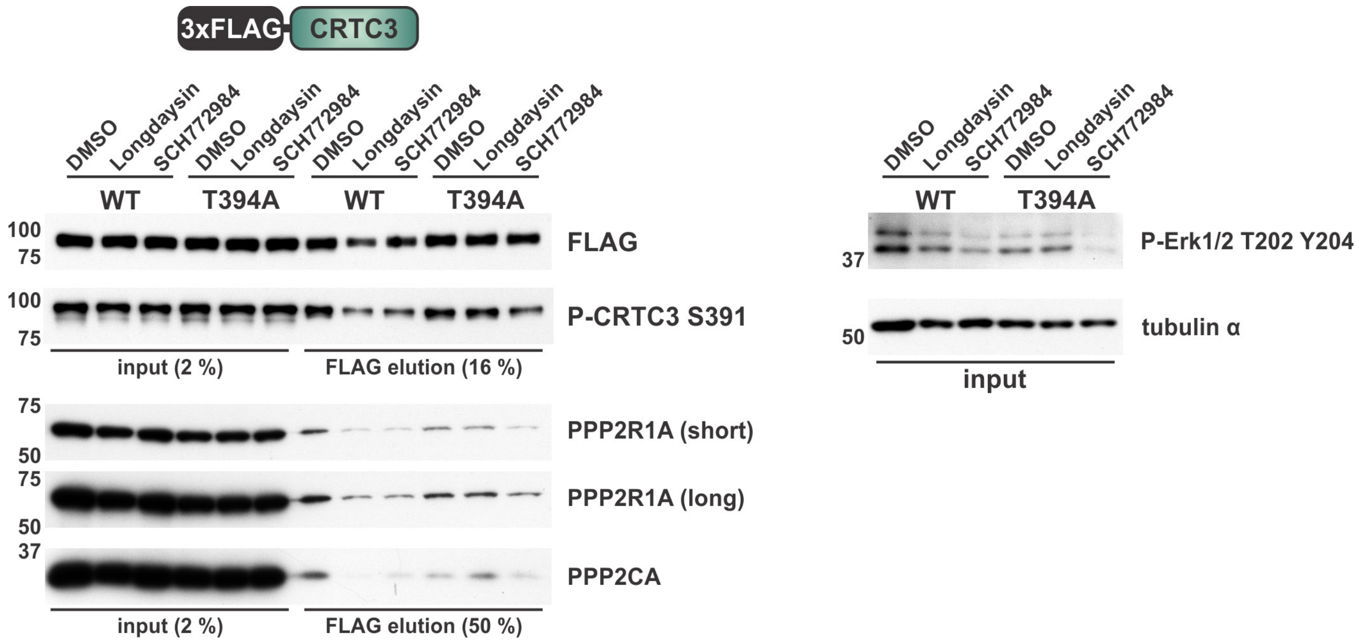

Fig. S6. The effects of Longdaysin on the CRTC3-PP2A complex formation requires the intact CK1 motif inside the PBR. Related to Fig. 5.

Western blot analysis of the Co-IP of FLAG-tagged CRTC3 WT and T394A mutant with endogenous PP2A. 4 h prior to the IP HEK 293T cells were treated with Longdaysin (20 µM; IC_{50} µM: CK1α/δ = 6-9, ERK2 = 52 (Hirota et al., 2010)) and SCH772984 (100 nM; IC_{50} µM: ERK1/2 = 0.004/0.001 (Morris et al., 2013)).

Fig. S7

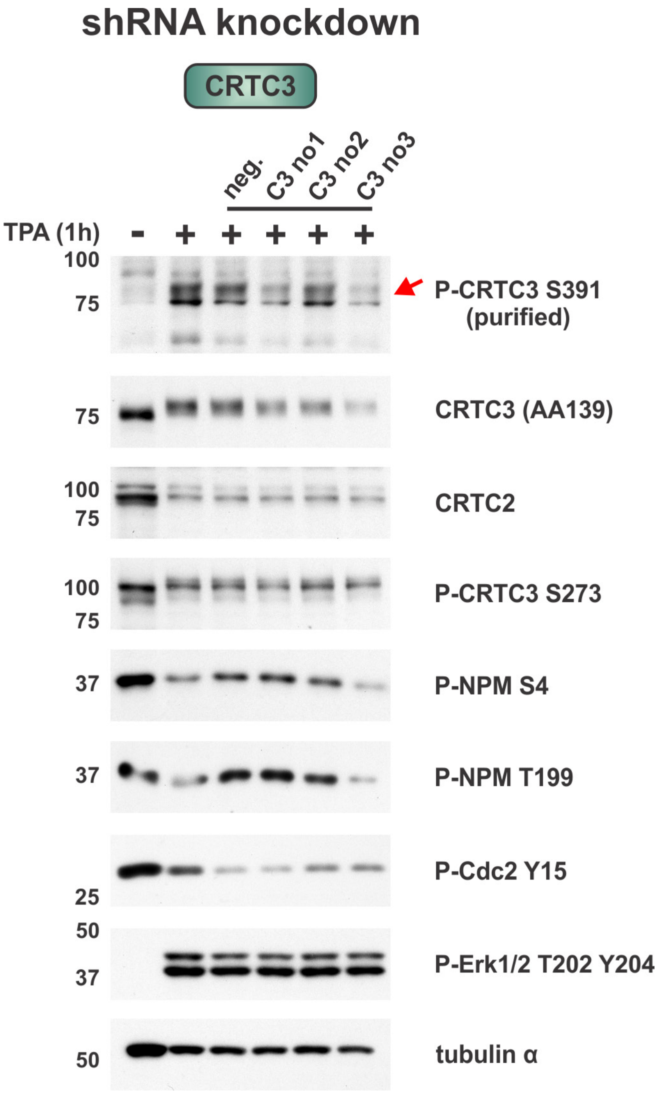

Fig. S7. P-CRTC3 S391 antibody evaluation. Related to Fig. 5.

A) Western blot analysis showing the effects of transient CRTC3 knockdown and TPA treatment (200 nM, 1 h) on S391 phosphorylation of endogenous CRTC3. HEK 293T cells were transfected with three different shRNA constructs against *H. sapiens* CRTC3 (C3 no 1-3; neg. = shRNA negative control). After 48 h cells were treated for 1 h with TPA (200 nM),

Transparent Methods

Sections of the methods were previously described and are reprinted here, partly verbatim, for reference (Sonntag et al., 2017).

Small molecules

Small molecules were solubilized in DMSO (ACS, Sigma-Aldrich) at the indicated concentrations and stored until usage at -80 ˚C (long term storage) or -20 ˚C (working dilution): 2 mM Carfilzomib (PR-171; Selleck Chemicals), 200 µM Cyclosporin A (cyclosporin; Sigma-Aldrich), 100 µM Dinaciclib (SCH727965; Selleck Chemicals), 20 mM Forskolin (Sigma-Aldrich), 20 mM Longdaysin (Sigma-Aldrich), 200 µg/ml nocodazole (Cell Signaling Technology), 1 mM Palbociclib (PD-0332991; freshly prepared in ddH_20; Selleck Chemicals), 20 mM Roscovitine (Seliciclib, CYC202; Selleck Chemicals), 100 µM SCH772984 (Selleck Chemicals), 400 µM TPA (Cell Signaling Technology).

Antibodies

The antibodies used in this study were purchased from Abcam (PPP2R1A), Covance (GFP), EMD Millipore (α-tubulin), Santa Cruz Biotechnology (14-3-3 ε, 14-3-3 ζ), Sigma-Aldrich (FLAG M2), and Cell Signaling Technology (14-3-3 [pan], Calcineurin A [CaN; pan], P-Cdc2 Y15, P-CREB S133, CRTC3 *H. sapiens* AA139 [#2720], P-CRTC2 S171, P-NPM S4, P-NPM T199, P-p44/42 MAPK [Erk1/2] T202/Y204, PPP2CA, PPP2R1A, PPP2R2A). The P-CRTC3 S273 antiserum was previously described (Sonntag et al., 2017).

See antiserum production for the rabbit P-CRTC3 S391 (PBL #7408), the rabbit hCRTC3(414-432) (PBL #7019), and the rabbit mCRTC2(454-477) (PBL #6896) antisera.

Antiserum production

Animal Care

All animal procedures were approved by the Institutional Animal Care and Use Committee of the Salk Institute

and were conducted in accordance with the PHS Policy on Humane Care and Use of Laboratory Animals (PHS Policy, 2015), the U.S. Government Principles for Utilization and Care of Vertebrate Animals Used in Testing, Research and Training, the NRC Guide for Care and Use of Laboratory Animals (8th edition) and the USDA Animal Welfare Act and Regulations. All animals were housed in an AAALAC accredited facility in a climate controlled environment (65-72 degrees Fahrenheit, 30-70% humidity) under 12-hour light/12-hour dark cycles. Upon arrival, animals were physically examined by veterinary staff for good health and acclimated for at least two weeks prior to initiation of antiserum production. Each animal was monitored daily by the veterinary staff for signs of complications and weighed every two weeks. Routine physical exams were also performed by the veterinarian quarterly on all rabbits.

For production of each antiserum (P-CRTC3 S391, mCRTC2(454-477) and hCRTC3(414-432)), three 10 to 12-week old, female New Zealand white rabbits, weighing 3.0 to 3.2 kg at beginning of the study, were procured from Irish Farms (I.F.P.S. Inc., Norco, California, USA). Rabbits were provided with ad libitum feed (5326 Lab Diet High Fiber), micro-filtered water and weekly fruits, vegetables and alfalfa hay for enrichment.

Preparation of Antigens

Synthetic peptides were synthesized in house or by RS Synthesis (Louisville, KY), HPLC purified to >95%, and amino acid sequenced verified by mass spectrometry. Peptides were covalently attached to large carrier proteins for use as immunogens. Cys^{383},$pSer^{391}$ mCRTC3(383-395)-NH_2, and Cys^{414} hCRTC3(414-432) were conjugated to maleimide activated Keyhole Limpet Hemocyanin (KLH) per manufacturer's instructions (Thermo Fisher Scientific). mCRTC2(454-477) was conjugated to bovine thyroglobulins via glutaraldehyde, 1% final, using reagents purchased from Sigma-Aldrich.

Specific peptides used to generate antisera are as follows:

Cys^{383},$pSer^{391}$ mouse CRTC3(383-395)-NH_2, CRRRQPPV(pS)PLTL-NH_2;

mouse CRTC2(454-477), KQFSPTMSPTLSSITQGVPLDTSK; and

Cys^{414} human CRTC3(414-432), CLAPYPTSQMVSSDRSQLS.

Injection and Bleeding of Animals

The antigen was delivered to host animals using multiple intradermal injections of peptide-KLH conjugate in Complete Freund's Adjuvant (initial inoculation) or incomplete Freund's adjuvant (booster inoculations) every three weeks. Rabbits were bled, <10% total blood volume, one week following booster injections and bleeds screened for titer and specificity. Rabbits were administered 1-2 mg/kg Acepromazine IM prior to injections of antigen or blood withdrawal. At the termination of study, rabbits were exsanguinated under anesthesia (ketamine 50 mg/kg and aceprozamine 1 mg/kg, IM) and euthanized with an overdose of pentobarbital sodium and phenytoin sodium (1 ml/4.5 kg of body weight IC to effect). After blood was collected death of animals was confirmed. All animal procedures were conducted by experienced veterinary technicians, under the supervision of Salk Institute veterinarians.

Characterization and purification of antisera

Each bleed from each animal was tested at multiple doses for the ability to recognize the synthetic peptide antigen; bleeds with highest titers were further analyzed by Western blot for the ability to recognize the full-length endogenous protein and to check for cross-reactivity with other proteins. Antisera with the best characteristics of titer against the synthetic peptide antigen, ability to recognize the endogenous protein, and specificity were affinity purified and used for all studies. Rabbit PBL #7408 anti P-CRTC3 S391, rabbit PBL #6896 anti-CRTC2, rabbit PBL #7019 anti-hCRTC3 were purified using Cys^{383},$pSer^{391}$mCRTC3(383-395)-NH_2, or mCRTC2(454-477), or Cys^{414} hCRTC3(414-432), respectively, covalently attached to Sulfolink agarose (Thermo Fisher Scientific) for cysteine containing CRTC3 peptides or to Affi-Gel 10 (BioRad) for the CRTC2 peptide with N-terminal lysine. Coupling of peptides to resins was per manufacturer's instructions. To ensure that the same batch of purified antibodies could be used for this and future studies, large volumes, 20 ml rabbit sera, from bleeds with similar profiles were purified.

Plasmids

For overexpression studies plasmids were used that contained the *H. sapiens* Ubiquitin C promoter (pUbC), whose activity is unaffected by cAMP signaling. The plasmids - pUbC-3xFLAG-TEVsite-His_6-MCS-IRESeGFP and pUbC-MCS-His_6-TEVsite-3xFLAG-IRESeGFP - have been described previously (MCS = multiple cloning site) (Sonntag et al., 2017).

CRTC1-3 and EGFP (Clontech) overexpression constructs were previously generated (Sonntag et al., 2017, Sonntag et al., 2018). The plasmids code for the following proteins (UniProt identifier): mCRTC1 (Q68ED7-1), mCRTC2 (Q3U182-1), and mCRTC3 (Q91X84-1) (m = *Mus musculus*).

The CRTC3 mutants were cloned by restriction enzyme digest, site-directed mutagenesis, fusion PCR (CRTC3 Δ369-379), inverse PCR (CRTC3 Δ330-364), and restriction-free (RF) cloning (CRTC2/3 hybrid proteins). Fusion PCR and RF cloning were performed as previously described (Sonntag and Mootz, 2011, Sonntag, 2017).

The CRTC3 shRNA constructs were generated by cloning 5' phosphorylated double stranded hCRTC3 oligonucleotides into the *Bam*HI and *Hin*dIII cut pSilencer 2.1-U6 puro plasmid (Applied Biosystems):

no. 1: 5´ GATCCGCTTCAGCAACTGCGCCTTTTCAAGAGAAAGGCGCAGTTGCTGAAGTTTTTTGGAAA 3´

no. 2: 5´ GATCCGAAGCTCCTCTGGTCTCCATTCAAGAGATGGAGACCAGAGGAGCTTCTTTTTTGGAAA 3´

no. 3: 5´ GATCCGCACATCAAGGTTTCAGCATTCAAGAGATGCTGAAACCTTGATGTGCTTTTTTGGAAA 3´

The empty plasmid served as negative control.

Cell culture

HEK 293T cells were purchased from ATCC (CRL-11268) and propagated in DMEM (Gibco®, high glucose) supplemented with 10% Fetal Bovine Serum (FBS; Gemini Bio-Products) and 100 U/ml penicillin-streptomycin (Corning Inc.). Primary stromal vascular fraction (SVF) cells of brown adipose tissue (BAT) were generated from C57BL/6 wild type mice and CRTC3 knockout mice (Yoon et al., 2018):

Interscapular BAT was collected and digested for 30 min in collagenase buffer (100 mM HEPES pH 7.4, 1.5 mg/ml collagenase I [Sigma C6885], 125 mM NaCl, 5 mM KCl, 1.3 mM $CaCl_2$, 5 mM D-glucose, and 2% BSA) with gentle shaking at 37°C. After digestion and centrifugation, SVF cells were separated from floating mature adipocytes. SVF cells were filtered and incubated in RBC lysis buffer (0.017 M Tris pH 7.4; 0.16 M NH_4Cl, and

0.01 M EDTA) for 10 min at room temperature. SVF cells were centrifuged, washed, plated, and ultimately propagated in DMEM supplemented with 10% FBS and 100 U/ml penicillin-streptomycin.

Overexpression & immunoprecipitation (IP)

Experiments were performed in 6 well plates by reverse transfecting HEK 293T cells (2.5×10^6 cells) with 2 µg plasmid DNA using Lipofectamine® 2000 (Invitrogen). 48 h post transfection cells were collected in PBS and resuspended in lysis buffer (50 mM Tris, 150 mM NaCl, 10% glycerol, 1% Igepal [Sigma-Aldrich], 1 mM DTT, EDTA-free cOmplete™ Protease Inhibitor Cocktail [Roche], Phosphatase Inhibitor Cocktail 2 and 3 [Sigma-Aldrich], 1 µM Carfilzomib; pH 8.0). The supernatant (= cell lysate) was either used in IP experiments or directly mixed with SDS-PAGE loading buffer. In all IP experiments, cells were pre-treated for 1 h with 1 µM Carfilzomib prior to cell lysis. Cell lysates were incubated with anti-FLAG® M2 magnetic beads and 3xFLAG peptide (100 µg/ml final) was used to elute bound proteins (both Sigma-Aldrich).

Immunoprecipitation & mass spectrometry (IP-MS)

The IP-MS protocol and data analysis of N- and C-terminally FLAG-tagged CRTC1-3 has been previously described (Sonntag et al., 2017).

Immunoprecipitation of endogenous CRTC3

Immunoprecipitation was performed from 4 x 100 mm dishes of HEK 293T cells (1.5×10^7 cells). 48 h post seeding cells were treated for 2 h with 200 nM SCH772984 as well as 1 h with 1 µM Carfilzomib and 200 nM TPA. Cell lysis was performed as described in the IP protocol. HEK 293T lysate was incubated with CRTC3 rabbit hCRTC3(414-432) (PBL #7019) antiserum immobilized on Protein A magnetic beads (Dynabeads®, Life Technologies) and washed three times with lysis buffer. Bound CRTC3 protein was eluted using a CRTC3 peptide (Tyr^{414} 415-432: YLAPYPTSQMVSSDRSQLS) resuspended in lysis buffer (100 µg/ml final).

Immunofluorescence

HEK 293T cells (0.75×10^6 cells) were plated in Poly-D-Lysine coated glass bottom dishes (MatTek Corporation) and under certain conditions reverse transfected with Lipofectamine® 2000 (Invitrogen) using 1 µg of mCRTC3 plasmid DNA (pUbC-3xFLAG backbone). 24 h post seeding/transfection, cells were either directly fixed with 4% paraformaldehyde or treated for 30 min with DMSO (0.05 % final), 10 µM Forskolin, 200 nM TPA prior to fixation. After incubation with primary antibodies (FLAG M2, PPP2R1A #2041, hCRTC3(414-432) PBL #7019), microscopy samples were incubated with secondary antibodies conjugated with Alexa Fluor® - 568 (goat anti-mouse / goat anti-rabbit) or in case of FLAG/PPP2R1A co-staining Alexa Fluor® - 568 and - 647 (568 - goat anti-rabbit & 647 - goat anti-mouse) (Life Technologies). Counterstaining with DAPI (Cayman Chemical Company) was performed before image acquisition (LSM 710; Carl Zeiss).

Luciferase reporter assays

Assays were performed in 96 well plates by reverse transfecting HEK 293T cells (100,000 cells). For each well 80 ng of DNA was used: 10 ng of EVX-Luc reporter plasmid (2x CRE half-sites, firefly luciferase) (Sonntag et al., 2017), 10 ng of FLAG-tagged CRTC1-3 plasmids, 60 ng of empty pUbC plasmid. 24 h post transfection 10 µM Fsk or 200 nM TPA were added and cells further incubated for 4 h (Fsk) or 5 h (TPA). Under all circumstances, DMSO served as the control treatment (each well 1% DMSO final). Next, 10 µl of Bright-Glo™ (Promega) was added per well and luciferase activity measured in a GloMax® multi microplate reader (Promega).

Gene expression analysis

CRTC3 WT and KO SVF cells were propagated to 60-80% confluency before (co-)treatment with 200 nM SCH772984 and DMSO (0.1 % final) for 4h and/or 10 µM Forskolin for 1h. Subsequently, cells were lysed in TRIzol® (Thermo Fisher Scientific) and RNAs extracted. cDNA was synthesized from 500 ng input RNA using First Strand cDNa synthesis kit (Roche) and quantified on a LightCycler® 480 II (Roche).

Primers used in quantitative real-time PCR (qPCR) experiments:

RPL32:

FP 5´ TCTGGTGAAGCCCAAGATCG 3'; RP 5´ CTCTGGGTTTCCGCCAGTT 3'

NR4A1:

FP 5´ CTCTGGTTCCCTGGACGTTA 3´; RP 5´ AGTACCAGGCCTGAGCAGAA 3´

RGS2:

FP 5´ AACGGCCCCAAGGTCGAGGA 3´; RP 5´ CGCTTCCTCAGGAGAAGGCTT 3´

PP2A activity assay

Experiments were performed in 6 well plates by reverse transfecting HEK 293T cells (2.5×10^6 cells) with 2 µg plasmid DNA using Lipofectamine® 2000 (Invitrogen). 48 h post transfection cells were treated for 1 h with 1 µM Carfilzomib, collected in PBS, and resuspended in lysis buffer (50 mM Tris, 150 mM NaCl, 10% glycerol, 1% Igepal [Sigma-Aldrich], 1 mM DTT, EDTA-free cOmplete™ Protease Inhibitor Cocktail [Roche], 1 µM Carfilzomib; pH 8.0). The supernatant (= cell lysate) was either used in the PP2A activity assay or directly mixed with SDS-PAGE loading buffer. PP2A catalytic subunit (PPP2CA) was immunoprecipitated from cell lysates according to manufacturer's instructions (PP2A Immunoprecipitation Phosphatase Assay Kit, Upstate). Phosphate release from 0.75 mM threonine phosphopeptide (KRpTIRR) was detected by Malachite Green and measured in a Synergy™ H1 microplate reader (BioTek Instruments).

Cell proliferation assay

Experiments were performed in 96 well plates by reverse transfecting HEK 293T cells (100,000 cells). In each well 80 ng of GFP or CRTC3 plasmid DNA was used. Effects on HEK 293T proliferation were measured using the BrdU Cell Proliferation ELISA Kit (Abcam) according to manufacturer's instructions in a Synergy™ H1 microplate reader (BioTek Instruments). BrdU treatment occurred either for 22 h (5 h post transfection) or for 4 h (23 h post transfection) prior to the colorimetric assay 27 h post transfection.

Sequence alignment

Amino acid sequences were aligned using MegAlign and Clustal W method (DNASTAR v7).

Statistical analysis

Data are either presented as the mean ± SEM. or ± SD. One-way ANOVA was used for qPCR and CRE reporter data analysis. Statistical analyses were performed using either Microsoft Excel (Microsoft Corporation) or PRISM (GraphPad). Graphical presentations were generated using PRISM (GraphPad) and SigmaPlot (Systat Software Inc.).

References

BEAUSOLEIL, S. A., VILLEN, J., GERBER, S. A., RUSH, J. & GYGI, S. P. 2006. A probability-based approach for high-throughput protein phosphorylation analysis and site localization. *Nat Biotechnol,* 24**,** 1285-92.

HIROTA, T., LEE, J. W., LEWIS, W. G., ZHANG, E. E., BRETON, G., LIU, X., GARCIA, M., PETERS, E. C., ETCHEGARAY, J. P., TRAVER, D., SCHULTZ, P. G. & KAY, S. A. 2010. High-throughput chemical screen identifies a novel potent modulator of cellular circadian rhythms and reveals CKIalpha as a clock regulatory kinase. *PLoS Biol,* 8**,** e1000559.

MORRIS, E. J., JHA, S., RESTAINO, C. R., DAYANANTH, P., ZHU, H., COOPER, A., CARR, D., DENG, Y., JIN, W., BLACK, S., LONG, B., LIU, J., DINUNZIO, E., WINDSOR, W., ZHANG, R., ZHAO, S., ANGAGAW, M. H., PINHEIRO, E. M., DESAI, J., XIAO, L., SHIPPS, G., HRUZA, A., WANG, J., KELLY, J., PALIWAL, S., GAO, X., BABU, B. S., ZHU, L., DAUBLAIN, P., ZHANG, L., LUTTERBACH, B. A., PELLETIER, M. R., PHILIPPAR, U., SILIPHAIVANH, P., WITTER, D., KIRSCHMEIER, P., BISHOP, W. R., HICKLIN, D., GILLILAND, D. G., JAYARAMAN, L., ZAWEL, L., FAWELL, S. & SAMATAR, A. A. 2013. Discovery of a novel ERK inhibitor with activity in models of acquired resistance to BRAF and MEK inhibitors. *Cancer Discov,* 3**,** 742-50.

SONNTAG, T. 2017. A Cassette Approach for the Identification of Intein Insertion Sites. *Methods Mol Biol,* 1495**,** 239-258.

SONNTAG, T. & MOOTZ, H. D. 2011. An intein-cassette integration approach used for the generation of a split TEV protease activated by conditional protein splicing. *Mol Biosyst,* 7**,** 2031-9.

SONNTAG, T., MORESCO, J. J., VAUGHAN, J. M., MATSUMURA, S., YATES, J. R., 3RD & MONTMINY, M. 2017. Analysis of a cAMP regulated coactivator family reveals an alternative phosphorylation motif for AMPK family members. *PLoS One,* 12**,** e0173013.

SONNTAG, T., VAUGHAN, J. M. & MONTMINY, M. 2018. 14-3-3 proteins mediate inhibitory effects of cAMP on salt-inducible kinases (SIKs). *FEBS J,* 285**,** 467-480.

YOON, Y. S., TSAI, W. W., VAN DE VELDE, S., CHEN, Z., LEE, K. F., MORGAN, D. A., RAHMOUNI, K., MATSUMURA, S., WIATER, E., SONG, Y. & MONTMINY, M. 2018. cAMP-inducible coactivator CRTC3 attenuates brown adipose tissue thermogenesis. *Proc Natl Acad Sci U S A*.
